# Excision of Four Inches of the Right Humerus Subsequent to Excision of the Head and Upper Third—Recovery with a Useful Arm

**Published:** 1866-09

**Authors:** A. J. Baxter

**Affiliations:** Chicago, Ill.


*Excision of Four Inches of the Right Humerus subsequent to Excision of the Head and Upper Third—Recovery with a Useful Arm.* By A. J. BAXTER, M. D., Chicago, Ill.

Excision of this articulation has been performed more or less during the last century, but the credit of bringing it prominently before the profession, and establishing its many advantages over what some would fain call the opprobrium of our art—amputation, is certainly due the surgery of the last ten years. Yet there are some points in connection with this operation which are to be determined by future experience—one of the most important of which is: How much of the superior extremity of the humerus can be removed, provided the soft parts and vessels are in a suitable condition, with a fair prospect of a useful arm remaining.

Mr. Guthrie, in speaking of gunshot injuries of the superior extremities, says: "An upper extremity should not be amputated for almost any accident which can happen to it from musket shot." I imagine if this holds good in regard to gunshot injuries, it will do for those produced by other agencies, as it is not the cause but the effect that necessitates surgical interference. But further on he would appear to throw some doubt on this assertion, where he says: "When the splinters extend far into the shaft of the humerus, it may be proper to amputate the whole extremity." Nearly all systematic writers, in speaking of this operation, say the incision should commence beneath the acromion and extend nearly as far as the insertion of the deltoid; and, as the incision is generally longer than the bone removed, it may be assumed that four inches is about the average maximum to which excisions have been carried, a greater amount of injury generally resulting in amputation. As a partial answer to this question, I submit the following case, for the notes of which I am indebted to my friend, Dr. W. C. Hunt:

"J. A——, fireman, aged 34, injured at a fire on the night of June 8th, 1865, by the falling of a brick wall. He was completely buried in the ruins; a portion of the wall striking the right shoulder (while he was standing) and another mass enclosing his body, so that his elbow rested upon it. The humerus was broken immediately below the head, and the splintered end of the lower portion protruded through the deltoid muscle and skin an inch or more. There was extensive contusion and laceration of the structures around the elbow joint. He was removed to his home, the end of the humerus excised, the head of the bone removed—making about four inches including the head of the bone. The wound at the elbow was dressed, and from that time he progressed apparently well until about the 1st of August, when it was discovered that the upper end of the bone was necrosed. At that time I was compelled to be absent from the city, when Dr. A. J. Baxter was requested to take charge of the case."

The arm was considerably enlarged, especially about the elbow, where quite a number of fistulous openings existed, leading up the arm; also a large one corresponding with the upper extremity of the humerus, through which dead bone could readily be detached. In consultation with Prof. J. W. Freer, it was decided to remove the dead bone—chloroform having been administered by Dr. Macdonald. An incision was made on the outside of the arm of sufficient extent to expose the upper extremity of the humerus, when it was discovered that the bone was much more extensively involved than was anticipated. About an inch and a half of the upper end was necrosed, and a line of separation commenced—below the bone was felt to be in a carious condition as far as the finger could be carried. Accordingly, the incision was extended to within a couple of inches of the elbow, and the dead and carious bone removed, making four inches—leaving about three inches of the lower extremity of the humerus. The periosteum was too much destroyed to be of any service in reproducing the bone. The wound healed without any trouble. A very severe eczema traumaticum made its appearance about the elbow and forearm, which was relieved by smearing the whole surface with ungt. zinci benzoatum and covering with oiled silk. The motions of the forearm and elbow are perfect. When the arm is hanging by the side, the hand can be raised to the breast. Although the elbow is gradually approaching the shoulder, and the movements of the arm improving accordingly, he probably will never be able to get the hand to the mouth.

